# Interaction between therapeutic interventions for Alzheimer’s disease and physiological Aβ clearance mechanisms

**DOI:** 10.3389/fnagi.2015.00064

**Published:** 2015-05-05

**Authors:** Christopher D. Morrone, Mingzhe Liu, Sandra E. Black, JoAnne McLaurin

**Affiliations:** ^1^Biological Sciences, Sunnybrook Research InstituteToronto, ON, Canada; ^2^Department of Laboratory Medicine and Pathobiology, University of TorontoToronto, ON, Canada; ^3^Canadian Partnership for Stroke Recovery, Sunnybrook Research InstituteToronto, ON, Canada; ^4^Department of Medicine (Neurology), University of TorontoToronto, ON, Canada; ^5^Hurvitz Brain Sciences Research Program, Sunnybrook Research Institute, Sunnybrook Health Sciences Centre and University of TorontoToronto, ON, Canada

**Keywords:** Alzheimer’s disease, amyloid-β peptide, clearance, vaccine, therapeutics, lifestyle factors

## Abstract

Most therapeutic agents are designed to target a molecule or pathway without consideration of the mechanisms involved in the physiological turnover or removal of that target. In light of this and in particular for Alzheimer’s disease, a number of therapeutic interventions are presently being developed/investigated which target the amyloid-β peptide (Aβ). However, the literature has not adequately considered which Aβ physiological clearance pathways are necessary and sufficient for the effective action of these therapeutics. In this review, we evaluate the therapeutic strategies targeting Aβ presently in clinical development, discuss the possible interaction of these treatments with pathways that under normal physiological conditions are responsible for the turnover of Aβ and highlight possible caveats. We consider immunization strategies primarily reliant on a peripheral sink mechanism of action, small molecules that are reliant on entry into the CNS and thus degradation pathways within the brain, as well as lifestyle interventions that affect vascular, parenchymal and peripheral degradation pathways. We propose that effective development of Alzheimer’s disease therapeutic strategies targeting Aβ peptide will require consideration of the age- and disease-specific changes to endogenous Aβ clearance mechanisms in order to elicit maximal efficacy.

## Introduction

Alzheimer’s disease (AD) is characterized in part by the accumulation of the amyloid-beta peptide (Aβ) within the brain parenchyma leading to cellular injury and ultimately death, as well as along blood vessels resulting in vascular dysfunction (Querfurth and LaFerla, 2010). It is suggested that the imbalance between Aβ production and clearance in aging drives Alzheimer’s disease progression in late onset Alzheimer’s disease (Hardy and Selkoe, 2002). In light of this, many clinical trials have been initiated over the last 20 years that have targeted removal or inhibition of Aβ production with very limited to no success.^1^ The failure of these trials has been attributed to targeting Aβ too late in the disease process, after the now recognized prodromal phase of Aβ accumulation in the absence of clinical symptoms (Sperling et al., 2011). Although these treatment strategies show high success rates in rodent models with amyloid precursor protein (APP) overexpression, a major confound is the lack of physiological deficits from aging and long term amyloid burden in these models, which are present in AD patients. Subsequently, endogenous Aβ degradation is diminished more prominently in AD than in disease models, a fact which is often overlooked in the development of treatments. This is a major contributing factor to the lack of clinical efficacy of Aβ specific therapies (Tanzi et al., 2004). We present three classes of AD treatments, and discuss how they rely on, and interact with, three physiological pathways of Aβ clearance. Firstly, we examine Aβ immunization strategies, and their dependence on the peripheral sink. Then we discuss the effectiveness of small molecule therapies targeting APP cleavage in the context of reduced Aβ degradation in the brain parenchyma. Finally, we present an array of lifestyle interventions in the clinic for AD, and discuss how these preserve vascular health, and how this may ultimately enhance interstitial fluid (ISF) and soluble Aβ drainage mechanisms. We survey therapeutic strategies that are presently in clinical development and describe the Aβ degradation pathways that are necessary for drug efficacy while highlighting potential disease-specific confounds that may contribute to previously failed trials.

## Immunization Strategies

Currently, there are both passive and active immunization strategies being developed for Alzheimer’s disease. These antibodies target various components of the AD pathology, such as Aβ and tau, but we will focus on those targeting Aβ (Table 1).

**Table 1:** **Therapeutic interventions presently under investigation for Alzheimer’s disease**.

Treatment name	Company	Therapy type	Ongoing clinical trial phase	Clearance mechanisms
Solanezumab (LY2062430)	Eli Lilly and Co	Passive immunotherapy	Phase 2/3 ongoing, Phase 3 ongoing	“Peripheral Sink Hypothesis” via LRP1 and ApoE, or astrocytes, endothelial cells, pericytes via PgP efflux pump across BBB, and subsequent phagocytosis by perivascular macrophages
Crenezumab (MABT51021A, RG7412)	Genentech	Passive immunotherapy	Phase 2 ongoing
CAD106	Novartis pharmaceuticals corporation	Active immunotherapy	None	FcRn-mediated IgG-assisted Aβ efflux across BBB with subsequent phagocytosis by perivascular macrophages + a non-Fc-mediated disruption of plaque structure
ACI-24 (Pal1-15 acetate salt)	AC immune SA	Active immunotherapy	Phase 1/2 ongoing
MK-8931 (MK-8931-09)	Merck	Small molecule	Phase 2/3 ongoing, Phase 3 ongoing	Suppression of Aβ production, so endogenous ADEs can degrade Aβ, such as: NEP, ECE-1, IDE, ACE, MMP-2, MMP-3, MMP-9, Plasmin
AZD3293 (LY3314914)	AstraZeneca	Small molecule	Phase 2/3 ongoing
VTP-37948	Vitae pharmaceuticals	Small molecule	Phase I ongoing
E2609	Biogen Idec, Eisai Co., Ltd.	Small molecule	Phase I and Phase II ongoing
TTP488 (PF-04494700)	Pfizer, TransTech Pharma, Inc.	Small molecule	Phase III pending	Suppression of Aβ transcytosis into the brain with endogenous ADEs to degrade Aβ within brain, such as: NEP, ECE-1, IDE, ACE, MMP-2, MMP-3, MMP-9, Plasmin

The most prominent area of immunization research has largely been focused on the development of antibodies against Aβ. One passive immunization antibody currently undergoing clinical trials is solanezumab [LY2064230]. It is the humanized IgG_1_ analog of the murine m266.2 antibody, an anti-Aβ monoclonal antibody (Samadi and Sultzer, 2011). *In vitro* studies have shown solanezumab to have a strong affinity (Kd of 10^−12^) towards the middle region of Aβ13-28 and thus acts primarily via a peripheral sink mechanism, although other physiological buffers and endogenous Aβ binding proteins may occur (DeMattos et al., 2001). The “peripheral sink” hypothesis is based on the premise that antibodies minimally transverse the blood-brain barrier (BBB) and thus clearance of Aβ from the brain relies on antibodies binding to Aβ within the bloodstream. Antibodies directed towards Aβ shift the balance of Aβ from the brain and the surrounding vasculature, leading to an efflux of Aβ into the periphery (DeMattos et al., 2001). However, in AD, this “peripheral sink” may be compromised, as Aβ efflux mechanisms may be less efficient or countered by influx of Aβ transcytosis into the brain (Kurz and Perneczky, 2011). Through rodent studies, it was shown that m266 treatment rapidly increased plasma Aβ40/42, which directly correlated with the amount of brain Aβ burden pre-treatment. These data support the idea that m266 acted as a “peripheral sink” to directly facilitate the efflux of Aβ from the brain (DeMattos et al., 2002). Another advantage of solanezumab is the selective binding to soluble Aβ, which greatly decreased the incidence of vasogenic edema and microhaemorrhages that were associated with earlier antibodies (Racke et al., 2005), called Amyloid Associated Imaging Abnormalities in human trials (Sperling et al., 2011). Phase 2 trials conducted in 52 mild-to-moderate AD patients in a double blind, placebo-controlled manner demonstrated a dose-dependent increase in plasma Aβ40 and Aβ42 as well as an increase in unbound Aβ42 in the CSF. These results suggested that solanezumab bound soluble Aβ, which thereby disrupted the equilibrium between soluble and insoluble Aβ within the CNS, resulting in reduction in the deposited Aβ burden (Farlow et al., 2012). Two Phase 3 trials, EXPEDITION-1 and -2 conducted in patients with mild-to-moderate AD did not show statistically significant improvement in cognition or activities of daily living as measured by the Alzheimer’s Disease Assessment Scales, (ADASCog 11 and 12) or the Alzheimer’s Disease Centers Scale for Activities of Daily Living (ADCS-ADL); however, there were significant differences in secondary outcome measures (Doody et al., 2014). Currently, there is a third Phase 3 trial in 2,100 mild AD patients who have elevated levels of Aβ plaques (EXPEDITION-3, ClinicalTrials.gov identifier: NCT01900665), a Phase 2/3 trial to test solanezumab in carriers of the APP, presenilin-1 and presenilin-2 Alzheimer’s gene mutations (DIAN Study, ClinicalTrials.gov identifier: NCT01760005), and a Phase 2 study in seniors deemed to be at high risk for AD who have amyloid positive PET scans (A4 Study, ClinicalTrials.gov identifier: NCT02008357).

Early studies, using passive immunization in AD patients, were often associated with microhemorrhages and vasogenic edema (Racke et al., 2005). In an attempt to avoid these side-effects, a humanized anti-Aβ monoclonal antibody with an IgG4 backbone called crenezumab (MABT5102A) was created (Adolfsson et al., 2012). Crenezumab is similar to solanezumab in that both target the midsection of Aβ; however, the IgG4 isotype reduces the risk of Fcγ receptor-mediated overactivation of microglial cells potentially leading to deleterious proinflammatory responses, while maintaining effective Aβ phagocytosis and clearance (Adolfsson et al., 2012). Furthermore, crenezumab recognizes both soluble Aβ oligomers and multiple Aβ aggregates that are present in AD brains (Adolfsson et al., 2012). *In vitro* experiments have shown that crenezumab both neutralized and protected neurons against toxic Aβ oligomers (Adolfsson et al., 2012). Phase 1 trials have shown to be extremely promising, as a dose-dependent increase in total plasma Aβ levels was observed to serum crenezumab concentrations demonstrating significant target interactions (Adolfsson et al., 2012). As well, no trial participants developed vasogenic edema, demonstrating the safety of high dose treatment (Adolfsson et al., 2012). Currently, there are several Phase 2 trials with crenezumab being tested as a method of prevention for carriers of the PSEN1 E280A mutations, (Alzheimer’s Prevention Initiative, ClinicalTrials.gov identifier: NCT01998841), on brain amyloid burden in mild to moderate AD (BLAZE Study, ClinicalTrials.gov identifier: NCT01397578) and a long-term safety extension study (ClinicalTrials.gov identifier: NCT01723826).

There has also been some success in terms of active Aβ immunotherapies for treatment of AD. Active Aβ immunotherapies are potentially more cost-effective and long-lasting compared to passive Aβ immunotherapies, which require recurring antibody infusions. Studies have shown an increase in cross-reactive, potentially protective Aβ autoantibodies as a result of active immunotherapy treatment in vervets, which are much lower in AD patients when compared to healthy, age-matched individuals (Weksler et al., 2002; Britschgi et al., 2009). However, active Aβ immunotherapies often require the use of a strong adjuvant for antibody production that may be detrimental to elderly patients who already exhibit an above average proinflammatory cytokine levels (Michaud et al., 2013b).

CAD106 is one such active immunization strategy that is currently in clinical trials. CAD106 was designed with multiple Aβ1-6 coupled to a virus-like Qβ particle, to avoid activation of inflammatory T cells (Wiessner et al., 2011). In APP transgenic mouse studies, CAD106 administration generated Aβ-specific antibodies without activation of Aβ-specific T cells (Wiessner et al., 2011). In prevention trials, CAD106 immunization significantly reduced plaque formation in APP24 mice, however in treatment trials of advanced plaque formation, efficacy was reduced. CAD106 treatment had no effect on levels of vascular Aβ and proinflammatory cytokines, and did not increase microhemorrhages. CAD106 treatment in rhesus monkeys, which share similar Aβ sequences to humans, showed a dose dependent increase in antibody production and Phase 1 trials in humans have found similar results (Wiessner et al., 2011; Winblad et al., 2012). Phase 1 trials showed that CAD106 did not have any adverse effects related to the treatment and a significant portion of the patients treated (67% in cohort 1 and 82% in cohort 2) developed Aβ antibody response that met the responder threshold (Winblad et al., 2012). Phase 2 trials were designed to establish antibody responses and tolerability to various doses, different regions of injection, and different doses of adjuvant. Partial results from Phase 2 findings have reported that long-term exposure to high amounts of CAD106 did not have any additional safety findings (Graf et al., 2014; ClinicalTrials.gov identifier: NCT01097096).

ACI-24 is another active immunization method under developed (Nicolau et al., 2002). ACI-24 is an Aβ1-15 peptide that is bound to liposomal surfaces through two palmitoylated lysine residues, forming a tandem at each end of the peptide (Muhs et al., 2007). The Aβ1-15 sequences were chosen as it retained the B cell epitope of Aβ, but lacked the T cell activation epitope (Monsonego et al., 2001, 2003). ACI-24 treatment restored memory deficits in mice, and produced mainly isotopes IgG, IgG2b, and IgG3, whereby the first two IgGs are related to noninflammatory Th2 response and the latter is a T cell-independent IgG subclass (Gavin et al., 1998; Muhs et al., 2007). Double transgenic mice treated with ACI-24 showed an improvement in memory function which correlated with an increase in IgG antibodies (Sigurdsson et al., 2004; Muhs et al., 2007). Furthermore, treatment resulted in significant reductions in both insoluble Aβ40 and Aβ42, as well as soluble Aβ42, with slight decreases in soluble Aβ40. This effect was observed without additional microglial activation, astrogliosis, or proinflammatory cytokine production (Muhs et al., 2007). Currently, there is a Phase 1/2 trial to examine the safety, tolerability, immunogenicity and efficacy of ACI-24 in mild-to-moderate AD patients (EudraCT Number: 2008-006257-40).

### Efflux Pathways for Clearance of Aβ

Since only 0.1% of all peripherally administered or *in vivo* generated antibodies cross the BBB, then the efflux of Aβ from the brain and subsequent degradation pathways in the periphery will be required for effective passive or active immunization strategies targeting Aβ (Banks et al., 2002; Morgan, 2011). The major efflux pathway for Aβ across the BBB is via the low density lipoprotein receptor-related protein -1 (LRP-1; Kanekiyo and Bu, 2014). LRP-1 is a large multi-functional receptor that regulates endocytosis of multiple ligands directly or indirectly through interaction with other ligands, such as Apolipoprotein E (ApoE), α2-macroglobulin, or other receptor associated proteins many of which have been implicated in AD pathogenesis (Liu et al., 2013; Kanekiyo and Bu, 2014). LRP-1 is abundantly expressed on neurons, glia and vascular cells within the brain and thus, is ideally located as a mechanism for Aβ clearance. For the present argument, LRP-1 is expressed on the microvascular including capillaries, venules and arterioles (Sagare et al., 2013). Furthermore, in AD patients and a mouse model of cerebrovascular amyloid angiopathy (CAA), LRP-1 staining is greatly reduced on vessels and is co-localized to amyloid plaques (Shibata et al., 2000; Deanne et al., 2004; Donahue et al., 2006). LRP-1 expression is also reduced in an age-dependent manner on microvasculature adding further to the potential deficits in Aβ efflux from the brain (Johanson et al., 2006). As mentioned, LRP-1 transports other ligands such as ApoE, the major lipid/protein chaperone in the brain which is known to bind directly to Aβ. There are 3 ApoE isoforms, ApoEε4 representing a risk factor for AD and having the lowest affinity for Aβ, ApoEε2 which represents a protective factor with a high binding affinity, and ApoEε3 which has an affinity between the two (Liu et al., 2013). Thus the differential affinity of the ApoE isoforms for Aβ binding could have further deleterious implications for the clearance of Aβ.

Studies on aging and AD have suggested that there is an increased permeability of the BBB with age and AD progression, as a result of oxidative stress and vascular changes, suggesting that passive diffusion of Aβ across the BBB might also contribute to Aβ efflux (Kalaria and Hedera, 1995; Marques et al., 2013). Under these conditions, a role for perivascular macrophages, pericyte or endothelial cell uptake, and degradation of Aβ after diffusion may also contribute (Verbeek et al., 2000; Hawkes and McLaurin, 2009). However, it has been proposed that macrophage function decreases with aging and pericytes are extremely sensitive to Aβ-induced toxicity, therefore the balance between these processes must be considered (Sengillo et al., 2013).

Lastly, the receptor for advanced glycation end-products (RAGE) plays an important role in AD by contributing to Aβ-induced neuronal dysfunction, microglial activation, and a key role in Aβ transcytosis into the brain (Yan et al., 1996, 2012; Deane et al., 2003; Origlia et al., 2008, 2010). In AD, Aβ can act as a ligand for RAGE and subsequently stimulate the upregulation of RAGE via a positive feed-back mechanism (Bierhaus et al., 2005). With RAGE-induced influx of Aβ into the brain, RAGE activity may negate the already compromised effects of Aβ efflux pathways, resulting in no change to the overall brain Aβ levels (Kurz and Perneczky, 2011). An oral inhibitor of RAGE, TPP488 (PF-04494700) has been shown to block these interactions. *In vitro* studies showed that TPP488 inhibited soluble RAGE binding to RAGE ligands, but more importantly in this context, to Aβ42.^2^ Phase 2 trials in people with mild-to-moderate AD showed that TPP488 was well tolerated in subjects that received either a low 10 mg or a high 20 mg dose (Sabbagh et al., 2011; ClinicalTrials.gov identifiers: NCT00566397, NCT00141661). However, results were inconclusive with respect to plasma Aβ levels and inflammatory markers, and there were no significant differences in cognitive and functional measures (Sabbagh et al., 2011). Recently, a Phase 3 trial of TPP488 in mild-to-moderate AD patients starting in 2014 was announced. The combination use of promoting Aβ clearance from the brain and blocking re-entry may provide a more powerful treatment then either strategy alone.

## Small Molecule Inhibitors

Currently, small-molecule inhibitors are being developed to inhibit various processes involved in Aβ plaque formation in AD. One class of inhibitors that are presently under examination by various studies are β-site APP Cleaving Enzyme (BACE) inhibitors. As β-secretase plays a major role in the production of Aβ peptides from APP, BACE inhibitors may reduce the amount of Aβ that is produced, allowing for endogenous Aβ clearance mechanisms to function more effectively. However, for these small-molecule inhibitors to work, they must cross both the BBB and enter the appropriate compartments within neurons (Vassar et al., 1999; Gabathuler, 2010). Therefore, in addition to target efficacy, BACE, these therapeutics must be developed with the appropriate molecular weight and charge to overcome these challenges (Pardridge, 2007). Specific BACE inhibitors are discussed below and summarized in Table 1.

MK8931, a small molecule inhibitor of BACE 1 and BACE2, has shown in Phase 1 trials that single doses of up to 500 mg were well tolerated and met with reductions in CSF Aβ of up to 92% in healthy individuals (Forman et al., 2012). Furthermore, MK8931 has shown to have a relatively long half-life, 20 h, which is ideal for single daily dosing paradigms (Forman et al., 2012; Stone et al., 2013). Currently, Phase 2/3 trials are examining the safety and efficacy of MK-8931 at daily dosages of 12 and 40 mg, as well as long-term treatment effects on ADAS-cog and ADCS-ADL scores (EPOCH, ClinicalTrials.gov identifier: NCT01739348 and APECS, NCT01953601).

AZD3293 (LY3314814) is an oral, brain permeable BACE 1 inhibitor, currently being developed by AstraZeneca and Eli Lilly (Haeberlein et al., 2013). Mouse and guinea pig studies have shown AZD3293 treatment resulted in a dose- and time-dependent reduction in the amount of Aβ40/42 and soluble β-APP in the brain, CSF and plasma (Haeberlein et al., 2013). Results from quantitative analyses of soluble α-APP and β-APP in healthy volunteers showed that AZD3293 treatment resulted in a dose-dependent decrease in the amount of soluble β-APP in the CSF, while soluble α-APP showed a similar broadly dose-dependent increase (Höglund et al., 2014). Phase 1 trials of AZD 3293 have recently been completed. A Phase 2/3 trial is currently recruiting, and will examine the safety and efficacy of the AZD 3293 over 2 years of treatment in early AD (ClinicalTrials.gov identifier: NCT02245737). Clinical Dementia Rating-Sum of Boxes (CDR-SOB) will be used as the primary outcome measure, with ADAS-COG and ADCS-ADL as secondary outcome measures, as well as other imaging and clinical markers.

Another oral BACE inhibitor, VTP-37948, currently in development by Vitae Pharmaceuticals, has shown good brain penetrance and reduced CSF Aβ levels by up to 80% in preclinical studies.^3^ At present, VTP-37948 is in Phase 1 trials with healthy individuals to examine safety and tolerability, as well as the pharmacokinetics and pharmacodynamics of the drug.

E2609 is a BACE1 inhibitor that is being developed by Eisai Ltd. It has been shown to significantly inhibit Aβ40/42 production in both the CSF and plasma of cynomolgus monkeys after oral dosing (Lucas et al., 2012). Partial results from Phase 1 studies have shown E2609 was well tolerated across all dosage treatments (up to 800 mg), and had a prolonged effect in reducing plasma Aβ40/42 after a single dosage in healthy individuals (Lai et al., 2012). Currently, trials have been completed on patients with MCI and those with evidence of Aβ pathology, and a dose-finding Phase 2 trial is underway in patients with MCI and mild AD (ClinicalTrials.gov identifier: NCT02322021). They also examined the safety and pharmacology of the E2609 across Japanese and Caucasian populations.

Further studies on BACE inhibitors must be conducted to determine efficacy in target engagement as well as potential off-target deleterious side-effects. Post-mortem analyses of AD brains demonstrated increased BBB permeability in brain regions, such as the hippocampus, which may aid BACE inhibitors to reach their targets (Montagne et al., 2015). However, >90% of AD patients exhibit CAA, amyloid deposition within the vasculature of the central nervous system, resulting in hypoperfusion as well as a physical barrier to influx of BACE inhibitors (Revesz et al., 2002; Thal et al., 2008). BACE cleaves many substrates, that play important roles in the nervous system, and thus inhibition of BACE may also lead to deleterious effects throughout the body. One example is neuregulin-1, a peptide that is essential for heart and nervous system development as well as the maintenance of muscle spindles (Britsch, 2007). Other BACE1 substrates, seizure-protein 6, L1, CHL1 and contactin-2, are important neural cell adhesion molecules that are crucial for guidance and maintaining neural circuits (Kuhn et al., 2012; Zhou et al., 2012). Furthermore, BACE1 KO mice demonstrate problems in axon targeting, although this may represent a developmental issue, re-programming of neural circuits and adult neurogenesis (Rajapaksha et al., 2011). Therefore, the consequences of total BACE inhibition on the critical function of these other substrates need to be examined further, as the effects of these BACE inhibitors on Aβ offers a promising therapeutic route. An alternate and possibly less prone to deleterious side-effects may be the development of modulators of BACE activity rather than inhibitors.

### Intraparenchymal Degradation Pathways

In order for small molecule therapies to be effective in AD, they not only need to exhibit high CNS bioavailability but also utilize endogenous parenchymal Aβ catabolism pathways. In regards to the BACE 1 inhibitors described above, these inhibitors will decrease new Aβ production and thus potentially prevent further neuronal and vascular damage. However, at time of treatment most AD patients will have a pre-existing Aβ load within the CNS that may require catabolism for full recovery. The endogenous parenchymal catabolic pathways for Aβ are regulated by a number of degrading enzymes in the extracellular space, secreted chaperones that monitor proteostasis, as well as glial cell uptake and degradation by lysosomal, autophagic and proteosomal pathways (Guénette, 2003; Tanzi et al., 2004; Lai and McLaurin, 2012; Wyatt et al., 2012).

Enzymatic degradation of Aβ peptides is accomplished by a number of enzymes including, but not limited to, neprilysin (NEP), insulin degrading enzyme (IDE), angiotensin converting enzyme (ACE) and various matrix metalloproteinases (MMPs). NEP is the most extensively studied, and has been shown to degrade both Aβ40 and Aβ42 *in vitro* and *in vivo* (Iwata et al., 2000, 2001). Furthermore, recent studies in aged mice and AD patients have shown decreased levels of NEP in the hippocampus and temporal gyrus, regions with a high amyloid load in AD (Yasojima et al., 2001; Iwata et al., 2002). However, the degradation of Aβ is complicated and cannot be accomplished by a single enzyme, as Kms and Aβ aggregation state will play a role. IDE has also been shown to have Aβ degrading activity, as IDE deficient mice have increased brain Aβ levels and over-expression in an AD mouse model decreases Aβ levels (Farris et al., 2003; Leissring et al., 2003). Furthermore, insulin competes with Aβ for IDE degradation and thus in Diabetes Mellitus, Aβ levels are increased within the CNS (Qiu and Folstein, 2006). The role of MMPs, ACE, endothelin-converting enzyme and others all contribute to Aβ catabolism however the precise role for each enzyme is not fully elucidated.

A recent hypothesis suggests that a family of secreted chaperones, which exist in the extracellular space, patrol the brain for misfolded proteins and aid in clearance (Wyatt et al., 2012). The chaperones relating to Aβ clearance that have been identified are clusterin (also referred to as ApoJ), α2-macroglobulin and ApoE. As mentioned above, all three chaperones are co-receptors for LRP-1 and thus aid in Aβ catabolism via uptake by neurons, glia or vascular cells.

In AD, astrocytes and microglia are the immune effectors of the CNS and thus play a role in injury resolution via limiting effects of toxic Aβ species (Guénette, 2003). Astrocytes become activated and surround amyloid plaques in an attempt to limit the damage to surrounding neuropil (Akiyama et al., 2000). Examination of human AD brain demonstrated the presence of N-truncated Aβ within astrocytes and more specifically Aβ was detected in lysosomal granules of astrocytes, thus suggesting phagocytosis and degradation (Funato et al., 1998; Thal et al., 1999; Nagele et al., 2003). In support of the pathological findings, adult mouse astrocytes have been shown to degrade Aβ deposits in brain sections, and phagocytose extracellular Aβ (Wyss-Coray et al., 2003; Koistinaho et al., 2004; Mandrekar et al., 2009). Although astrocyte uptake and degradation is less efficient then resident microglial cells, this pathway may contribute to Aβ clearance under treatment strategies.

Microglial cells are the resident phagocytes of the CNS and play a significant role in Aβ clearance (reviewed in Lai and McLaurin, 2012). Although microglial cells *in vitro* readily phagocytose and degrade soluble and fibrillary Aβ, there is some controversy regarding efficacy under the pathological conditions present in AD brains (Lee and Landreth, 2010). Similar to astrocytes, microglia surround amyloid plaques, and electron microscopy studies have suggested the intracellular presence of Aβ in endosomal compartments (Frackowiak et al., 1992). Furthermore, Aβ can be detected within lysosomal compartments of non-plaque associated microglial cells after treatment with an anti-aggregant compound, 1-fluoro-*scyllo*-inositol (Hawkes et al., 2012). Thus, although some literature suggests that accumulation of Aβ and amyloid plaques may be the result of immuno-incompetent microglia in AD, the presence of small molecules therapies, such as immunotherapy, curcumin and *scyllo*-inositol, that boost phagocytosis support a role for microglia in therapeutic interventions (Wilcock et al., 2004; McLaurin et al., 2006; Yanagisawa et al., 2010).

## Lifestyle Interventions

In recent years, AD research has begun to focus on changes that can be made in daily living and activity which potentially decrease risk and delay symptomatic expression of AD. During the International Conference on Nutrition and the Brain a compilation of the presented data led to the identification of 7 changes to be integrated into daily living, six involving diet and the seventh recommending exercise (Barnard et al., 2014). Exercise and diet alterations have been associated with improvements in symptomatic and pathophysiological AD outcomes in both animal models and humans (Luchsinger et al., 2002; Stranahan et al., 2012; Hawkes et al., 2015; Lim et al., 2015; Lin et al., 2015). Regular, controlled diet and exercise are likely protective against AD pathogenicity through the maintenance of cardiovascular and cerebrovascular health. As mentioned above, the vasculature plays a crucial role in the clearance of Aβ across the BBB because of receptors (i.e., LRP-1, RAGE) expressed on the plasma membranes of capillary, arteriole, and small venule cells (Deane et al., 2003; Sagare et al., 2013). It has recently become apparent that regular and healthy pulsation of blood vessels promote a convective bulk flow of the brain’s parenchymal ISF, which acts to clear toxic solutes such as Aβ (Carare et al., 2008; Weller et al., 2008; Iliff et al., 2012). Lifestyle interventions, including exercise, diet and sleep, may have direct beneficial effects on Aβ clearance, as well as act indirectly through enhancing vascular health.

### Exercise

There are >20 ongoing and recently completed clinical trials assessing the efficacy of different exercise interventions on AD symptoms and pathology.^4^ Thoroughly detailing these trials is beyond the scope of this review, however a few points will be noted. Firstly, exercise has had beneficial effects on many cognitive assessments (de Andrade et al., 2013; Winchester et al., 2013; Okonkwo et al., 2014). Secondly, AD interventional benefits are seen in a multitude of exercises ranging from walking to high-intensity aerobic physical activity (Venturelli et al., 2011; Nascimento et al., 2012; Vidoni et al., 2012; Hoffmann et al., 2013; Suttanon et al., 2013; Winchester et al., 2013; Arcoverde et al., 2014; Okonkwo et al., 2014). Finally, regular exercise over years is correlated with significantly slowed Aβ deposition over time, perhaps in part due to enhanced Aβ clearance (Okonkwo et al., 2014).

The beneficial effects of aerobic exercise on brain health have been well studied in recent years, with a focus on enhanced neurogenesis, increased levels of neurotrophic factors such as brain-derived neurotrophic factor (BDNF), and reduced risk for AD (Voss et al., 2013). Lin et al. (2015) demonstrated that exercise is associated with improved Aβ clearance mechanisms by upregulation of LRP-1 protein levels whereas RAGE was unchanged (Lin et al., 2015). Interestingly, one study in the Tg2576 AD mouse model demonstrated reduced soluble Aβ, but unchanged total Aβ, as a result of exercise (Nichol et al., 2008). This further suggests that Aβ clearance mechanisms specifically are upregulated with exercise. BDNF signaling in the brainstem of mice has recently been shown to increase the excitability of parasympathetic cholinergic neurons which, by way of the vagal nerve, act to lower resting heart rate (Wan et al., 2014). This provides molecular evidence for a mechanism by which aerobic exercise can reinforce vascular health (Wan et al., 2014; Mattson, 2015). Since the structurally and functionally impaired cerebrovasculature in AD dampens blood vessel-dependent Aβ clearance pathways, physical activities which can upregulate BDNF signaling and provide enhanced amyloid removal to the periphery, would be beneficial in slowing disease progression (Dorr et al., 2012; Lai et al., 2015).

### Diet

Healthy diet maintenance is important in the modulation of AD symptoms and pathology, assisting endogenous mechanisms including neurogenesis, antioxidant protection, and Aβ clearance (Aliev et al., 2013; Maruszak et al., 2014). Type 2 Diabetes Mellitus (T2DM) is an acquired disease caused by hyperglycaemia and potentially insulin resistance. The main risk factors for the incidence and prevalence of T2DM are obesity, primarily from high fat diets, and age (Barbagallo and Dominguez, 2014; Centers for Disease Control and Prevention, 2014). Studies have shown that T2DM patients are at an increased risk of developing dementia, and conversely, 80% of AD patients have T2DM (Janson et al., 2004; Biessels et al., 2006). In a recent study by Hawkes et al. (2015) mice that were subject to a high fat diet during gestation and early life exhibited deficits in perivascular clearance of Aβ, an effect which was exacerbated when the high fat diet was lifelong. Also, vascular deposits of Aβ in aged human cases of hyperlipidemia were significantly greater when compared to aged-matched people with normal lipid levels post-mortem (Hawkes et al., 2015).

Dietary plans have been well researched in AD. The main three are the Mediterranean, ketogenic, and caloric restriction diets (Aliev et al., 2013; Maruszak et al., 2014). The Mediterranean diet involves replacement of meat products, especially red meat, with plant-based alternatives, and primarily olive and fish oils for additional fats (Yannakoulia et al., 2015). Clinical studies on the effect of this diet on cognition in MCI/AD are inconsistent, with some showing benefits and others not (Olsson et al., 2015; Yannakoulia et al., 2015). Contrary to the Mediterranean diet, ketogenic and caloric restriction diets involve a significant reduction in food intake (Maruszak et al., 2014; Paoli et al., 2014). The ketogenic diet aims to create a state of fasting within the body (Paoli et al., 2014). This reduces metabolic induced stresses, including damage from reactive oxidative species and pathogenic mitochondrial biogenesis (Paoli et al., 2014). Ketogenic diets may also decrease the production of advanced glycation end products, which accumulate on Aβ plaques, potentially assisting in one of the aforementioned clearance cascades by decreasing reuptake of Aβ by RAGE (Deane et al., 2003; Srikanth et al., 2011; Paoli et al., 2014). Caloric restriction on the other hand, is achieved by a moderate decrease in overall intake of calories (Maruszak et al., 2014). Caloric restriction has been associated with a reduced risk of AD and memory improvements in the elderly, and has been a beneficial intervention in mouse models of AD (Lee et al., 2000, 2002; Luchsinger et al., 2002; Gustafson et al., 2003; Wu et al., 2008; Witte et al., 2009). This diet has also been demonstrated to decrease Aβ pathology through enhancing non-amyloidogenic APP cleavage by α-secretase and increasing clearance of Aβ through upregulated IDE levels (Farris et al., 2004; Wang et al., 2005; Tang and Chua, 2008). There are two current clinical trials on the effectiveness of caloric restriction in MCI/AD.

### Sleep

Sleep and circadian rhythm disturbances are common in aging and AD patients (Floyd et al., 2000; Cipriani et al., 2014; Zelinski et al., 2014). Circadian rhythms are controlled by the suprachiasmatic nucleus (SCN) in the hypothalamus, which acts like a biological clock in its management of many physiological functions (Reppert and Weaver, 2001; Coogan et al., 2013; Videnovic et al., 2014; Zelinski et al., 2014). Mouse models of AD exhibit circadian rhythm alterations suggesting a link to Aβ (Sterniczuk et al., 2010; Baño Otalora et al., 2012). Even self-reported disruptions in sleep indicate a 33% increased risk of dementia and a 51% increased risk of AD (Benedict et al., 2014). Higher brain amyloid burden and lower CSF Aβ levels were observed in sleep deprived and narcoleptic patients without AD (Spira et al., 2013; Liguori et al., 2014). AD animal models in which sleep is deprived show similar trends of increased amyloid load, as well as increased memory dysfunction (Kang et al., 2009; Rothman et al., 2013; Di Meco et al., 2014).

A study in cognitively normal middle-aged men measured CSF biomarkers of AD by an intrathecal catheter (Ooms et al., 2014; AWAKE study, ClinicalTrials.gov identifier: NCT01194713). A 6% decrease in Aβ42 levels were observed following an unrestricted sleep, however no change was observed in participants who remained awake (Ooms et al., 2014). The benefits of sleep on soluble Aβ levels in the brain may have been underestimated due to measurement of only spinal CSF (Ooms et al., 2014). There is also enhanced clearance by the glymphatic system during sleep, leading to additional Aβ efflux into the blood and cervical lymph nodes (Szentistványi et al., 1984; Iliff et al., 2012; Xie et al., 2013). Using *in vivo* two-photon microscopy in sleeping and in anesthetized mice, Xie et al. (2013) measured a 60% increase in the brain parenchyma, associated with an increase in the rate of exchange of CSF-ISF. These results suggest that regular sleep may serve to aid in the clearance of toxic solutes, such as Aβ, from the extracellular space in the brain, and normalization of sleep patterns may serve as beneficial lifestyle intervention in the treatment of AD (Xie et al., 2013).

There are 7 active clinical trials for sleep interventions in AD, two of which are directly concerned with sleep apnea. Sleep apnea causes pauses or disruptions in breathing during sleep, and is correlated with cognitive dysfunction in AD patients (Janssens et al., 2000; National Heart, Lung, and Blood Institute, 2012). It is potentially associated with AD through mechanisms such as cellular oxidative stress, hypoxia, and sleep disturbances (Pan and Kastin, 2014). The two current clinical trials for sleep apnea and AD are assessing the effectiveness of a continuous positive airway pressure (CPAP) device on measurements including cognition, quality of daily living and CSF levels of Aβ42 (AZAP, ClinicalTrials.gov identifier: NCT01400542 and SNAP, ClinicalTrials.gov identifier: NCT01962779). The use of CPAP has effectively improved sleeping conditions in patients with mild-to-moderate AD, an effect that was maintained for 3 weeks (Cooke et al., 2009).

Sleep apnea leads to impaired vascular health, with increased arterial stiffness and blood pressure, decreased cerebral blood flow, and thickened carotid intima-media (Daulatzai, 2012; Ciccone et al., 2014). The tunica intima and tunica media are the innermost perivascular layers along cerebral arteries, and the tunica media is the area within which the majority of ISF flows on its way out of the brain during perivascular clearance (Carare et al., 2008; Weller et al., 2008, 2010). Therefore if sleep disturbances are thickening this area, the rate at which bulk flow of ISF travels would theoretically be decreased. This, in conjunction with the impaired arterial health in sleep apnea, would potentially diminish the perivascular clearance of Aβ, leading to increased CAA and amyloid in the brain parenchyma (Daulatzai, 2012). Nighttime wakefulness also leads to a decrease in glymphatic Aβ clearance because of the inability for parenchymal expansion, which normally speeds the rate of CSF-ISF exchange during sleep (Xie et al., 2013). Both glymphatic and perivascular clearance mechanisms are further diminished by the impaired vasculature present in sleep disorders due to irregular blood vessel pulsations (Carare et al., 2008; Iliff et al., 2013b).

### Vascular Health

Hypertension, increased body mass index, abnormal glucose regulation, hyperlipidemia, and hypercholesterolemia are all vascular related risk factors for AD and cognitive decline (Kivipelto and Solomon, 2008; Reynolds et al., 2010; Tolppanen et al., 2012; Liu et al., 2014; Deckers et al., 2015). AD patients analyzed for the relationships between cardiovascular risk factors and cognition showed lower scores on the MMSE and ADAS-COG in hypertensive patients compared to those with normal blood pressure, and lower scores on the MMSE and Clinical Dementia Rating in patients with hyperlipidemia compared to those without (Lobanova and Qureshi, 2014). Such findings suggest vascular injury may play a role in cognitive dysfunction in AD. Vasoactive drugs therefore might have the potential to treat aspects of AD, including deficits in cognition. Two current vasoactive drugs in development for AD are nilvadipine and cilostazol (Ikeda, 1999; Nimmrich and Eckert, 2013). Nilvadipine is a dihydropyridine that blocks calcium channels and prevents cognitive decline in patients with MCI (Hanyu et al., 2007; Nimmrich and Eckert, 2013). Although intervention with Nilvadipine leads to decreased hypertension, its main effect on cognition is postulated to be through neuroprotection, potentially by decreased calcium-mediated excitotoxicity of neurons (Takakura et al., 1992; Hanyu et al., 2007; Nimmrich and Eckert, 2013). A Phase 3 placebo-controlled trial of nilvadipine in mild-moderate AD is underway (NILVAD, ClinicalTrials.gov identifier: NCT02017340).

Cilostazol has multiple beneficial effects on the vasculature, antiplatelet activity, and is an inhibitor of the cAMP and cGMP regulator phosphodiesterase type 3 (PDE3; Ikeda, 1999; Saito and Ihara, 2014). Studies of cilostazol in human dementia/AD patients showed some benefit on slowing cognitive decline and increasing cerebral perfusion (Sakurai et al., 2013; Ihara et al., 2014). Patients with mild dementia, but not moderate to severe dementia, maintained higher MMSE scores when treated with cilostazol in conjunction with the acetylcholinesterase inhibitor donepezil, compared to donepezil alone (Ihara et al., 2014; CASID study, ClinicalTrials.gov identifier: NCT01409564). Slower decline in ADAS-COG scoring was also seen in another study, along with increased regional cerebral blood flow to the right anterior cingulate lobe (Sakurai et al., 2013). Cilostazol is theorized to slow AD progression by promoting perivascular clearance of ISF, which contains soluble Aβ, in part due to it vasodilation and regulation of blood vessel pulsations (Carare et al., 2008; Han et al., 2013; Saito and Ihara, 2014). Additionally, PDE3 expression is increased in arterial cells with Aβ deposition, primarily in smooth muscle cells (Maki et al., 2014). Smooth muscle cells are present within the tunica media of the perivascular space along leptomeningeal arteries, a pathway through which ISF, including soluble Aβ, is cleared from the brain (Weller, 2005; Kwee and Kwee, 2007; Carare et al., 2008; Weller et al., 2008). In a mouse model of CAA, cilostazol protected against vascular and cognitive deficits, and decreased Aβ deposits, potentially because of enhanced perivascular clearance (Maki et al., 2014). This suggests beneficial effects of cilostazol in AD on perivascular clearance of ISF, and on a vascular protective signaling cascade involving inhibition of PDE3 and increased cAMP and cGMP activity.

### Perivascular Clearance

**Figure 1:**
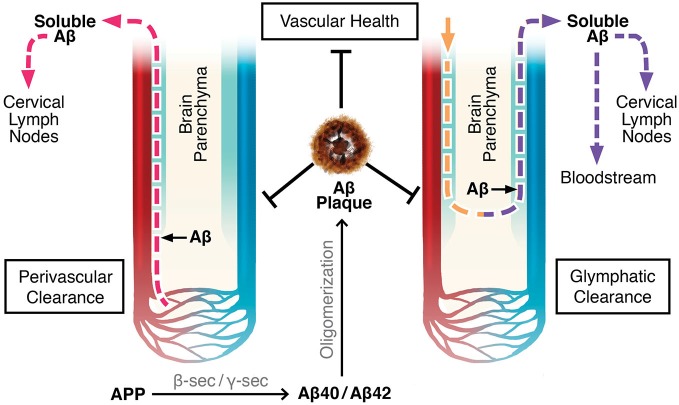
**Perivascular and glymphatic drainage of brain interstitial fluid (ISF)**. These mechanisms drain brain ISF and parenchymal solutes (e.g., Aβ) to the periphery. Perivascular clearance involves ISF efflux along capillaries, arterioles, and arteries within the perivascular space. Glymphatics involve CSF influx and CSF/ISF efflux within the perivascular space along arteries and veins, respectively. **Color Legend**, red: arteries/arterioles/capillaries; blue: veins/venules; light blue: perivascular space; pink dotted line: ISF efflux; yellow dotted line: CSF influx; purple dotted line: CSF/ISF efflux.

There are two proposed mechanisms by which drainage of the brain’s extracellular fluid, also referred to as ISF, occur (Carare et al., 2008; Iliff et al., 2012). The first involves a bulk flow of ISF and solutes within arterial perivascular spaces, across the BBB, where they enter cervical lymph nodes (Szentistványi et al., 1984; Carare et al., 2008). Perivascular spaces, or Virchow-Robin spaces, are small areas around blood vessel walls, including the smooth muscle cells which line arteries, that are in continuity with the subarachnoid space from the point where blood vessels penetrate into the brain parenchyma (Weller, 2005; Kwee and Kwee, 2007; Weller et al., 2008). The pia mater sheaths the perivascular space along leptomeningeal arteries until they branch off into smaller arterioles and capillaries (Weller, 2005; Kwee and Kwee, 2007; Carare et al., 2008). According to one theory, flow of ISF occurs along the basement membranes of capillaries and arterioles until it converges upon leptomeningeal arteries, where the fluid continues to drain in the perivascular tunica media, the space between the arterial smooth muscle cells, and in the tunica adventitia, the basement membranes of the smooth muscle cells (Carare et al., 2008; Weller et al., 2008, 2010). There is further convergence with leptomeningeal and major cerebral arteries before the ISF drains completely out of the brain into the cervical lymph nodes (Szentistványi et al., 1984; Weller et al., 2010). This theory implies that flow of ISF occurs retrogradely only along capillaries, arterioles, and arteries, not along venules or veins (Figure 1; Carare et al., 2008; Arbel-Ornath et al., 2013).

Evidence for perivascular clearance has been obtained by experiments involving radio- or fluorescent labeled tracers. Furthermore, multi-photon imaging and mathematical models have provided strong support (Szentistványi et al., 1984; Schley et al., 2006; Carare et al., 2008; Wang and Olbricht, 2011; Arbel-Ornath et al., 2013). ISF drainage was theorized to be by a convective bulk flow mechanism by assessing the clearance of multiple radiolabeled compounds of differing molecular weights. These compounds cleared at the same rate, confirming that ISF did not drain by diffusion, in which case the smaller molecules would have moved faster (Cserr et al., 1981; Abbott, 2004; Carare et al., 2008). Using multi-photon imaging through a surgically dissected cranial window in mice, Arbel-Ornath et al. (2013) observed active perivascular clearance of ISF *in vivo*. Consistent with previous conclusions, the fluorescent tracer flowed out of the brain along capillaries and arteries but not veins (Arbel-Ornath et al., 2013). Perivascular clearance is reliant on proper vasculature flow, as it is non-existent after cardiac arrest and is diminished with decreased perfusion (Carare et al., 2008; Arbel-Ornath et al., 2013). Theoretical models propose that the pulsating motion of blood flowing into the brain acts to push ISF in the opposing direction, thereby explaining why the draining of ISF requires functioning vasculature, and why fluid is not cleared along veins (Schley et al., 2006; Carare et al., 2008; Wang and Olbricht, 2011).

Although Aβ self-aggregates into fibers and forms plaques, there is still a significant portion of soluble species of Aβ that is released to the extracellular space (Selkoe, 2001). Since convective bulk flow of ISF drains solutes, perivascular drainage represents one of the major Aβ clearance mechanisms and could be impaired in AD (Carare et al., 2008). The APP/PS1 AD mouse model showed a 60% increased retention of fluorescent tracer, compared to wild type mice (Arbel-Ornath et al., 2013). The presence of Aβ plaques on arteries cause increased tortuosity and decreased calibre which in turn disrupts and lengthens blood flow transit times (Dorr et al., 2012). This irregularity in the vasculature is likely interrupting the pulsating motion which drives perivascular clearance. Also, it was found that fluorescent conjugated tracers and Aβ40 when injected are present where plaques would form in AD (Hawkes et al., 2013, 2014). The hippocampus of non-transgenic mice showed an aging-related decrease in perivascular clearance of injected Aβ40, suggesting the importance of this clearance mechanism to AD pathogenesis (Hawkes et al., 2013). Together, these conclusions support a positive feedback loop for CAA and AD, where impaired perivascular ISF drainage leads to Aβ deposition along the arteries, which causes weakened vasculature functioning, thereby allowing increased levels of Aβ to remain in the brain parenchyma, more specifically in the hippocampus (Carare et al., 2008; Hawkes et al., 2013).

### Glymphatics

The glymphatic system is another ISF drainage system recently proposed by Maiken Nedergaard’s group (Iliff et al., 2012; Iliff and Nedergaard, 2013). Glymphatics involve the para-arterial, unidirectional flow of CSF from the cisterna magna of the subarachnoid space, along penetrating leptomeningeal arteries, towards the pituitary and pineal recesses of the 3rd ventricle, and into the brain within the perivascular (Virchow-Robin) space (Iliff et al., 2012, 2013a). CSF enters the brain parenchyma and mixes with ISF in a process reliant on aquaporin-4 (AQP4) channels present on the perivascular endfeet of astrocytes. The extracellular fluid composed of CSF and ISF is proposed to exit the parenchyma by drainage out of the brain within the venous perivascular space of large calibre veins only, where it then flows into the cervical lymph nodes, or absorbs into the blood across arachnoid villi on dural sinuses (Figure 1; Szentistványi et al., 1984; Iliff et al., 2012). Similar to perivascular clearance, glymphatic flow is driven by the pulsation of blood vessels; however, the former is by bidirectional flow along the vasculature, and the latter is unidirectional (Hadaczek et al., 2006; Schley et al., 2006; Wang and Olbricht, 2011; Iliff et al., 2013b).

The glymphatic system was observed *in vivo* in mice after an intrathecal injected contrast agent was followed by magnetic resonance imaging, and through a closed cranial window with two-photon laser scanning microscopy (Iliff et al., 2012, 2013a). These results were reproduced using clinically-relevant levels of intrathecal injections in rats and mice (Yang et al., 2013). In confirmation that arterial pulsations drive the unidirectional flow of CSF into the parenchyma, unilateral ligation of the mouse carotid artery decreased blood flow and pulsality as well as flow of the injected tracers. Subsequently, treatment with dobutamine to increase blood flow and pulsality, increased tracer movement (Iliff et al., 2013b).

Similar to perivascular clearance, soluble Aβ is cleared from the brain parenchyma by glymphatic drainage (Carare et al., 2008; Iliff et al., 2012). Interestingly, the rates of CSF-ISF exchange significantly decrease with age, suggesting an age-related decline in the glymphatic clearance of toxic solutes, such as Aβ (Kress et al., 2014). Unlike perivascular clearance, the physics of the overall drainage system are affected by molecular size, because larger tracers were slower to enter the parenchyma following subarachnoid injection, suggesting a reliance on AQP4 channels for CSF-ISF exchange (Iliff et al., 2012). In healthy conditions, the expression of AQP4 is highly polarized to astrocytic endfeet (Iliff and Nedergaard, 2013). However, with neuroinflammation as well as with age, this expression profile changes, with decreased AQP4 at the perivascular endfeet and increased levels in the soma. This may explain the impairment in glymphatic flow with age, suggesting another mechanism by which Aβ clearance may be diminished in AD (Iliff and Nedergaard, 2013; Kress et al., 2014). Additionally, both the perivascular and the glymphatic clearance systems are impaired following an ischaemic stroke in mice (Arbel-Ornath et al., 2013; Gaberel et al., 2014). Because of a close clinical relationship with stroke, this provides further evidence to why drainage of Aβ may be impaired in AD (Viswanathan et al., 2009).

In addition, late-onset AD is thought to relate to impaired cerebral clearance of Aβ (Mawuenyega et al., 2010). This is in contrast to overproduction which leads to amyloid accumulation from mutations affecting APP processing (e.g., mutations in APP or presenilin complex), resulting in early-onset Autosomal Dominant AD or trisomy 21, which predisposes to early-onset dementia in Down’s syndrome. In this context, collagenosis of the deep penetrating venular system could be predicted to affect the glymphatic clearance of amyloid and other toxic proteins along the perivascular venular pathways. Collagenosis causes wall thickening and stenosis of the deep venular system and correlates with confluent periventricular white matter hyperintensities (pvWMH), which is seen in elderly controls and Alzheimer’s patients on Magnetic Resonance images (Moody et al., 1995; Black et al., 2009). PvWMH are associated with hypertension and other vascular risk factors, older age and possible genetic factors. Recent work from our group suggests the correlation of pvWMH is strongest with significant stenosis of the medium to large venules. We hypothesize that this leads to BBB leakage and perivenous edema, which further disrupts clearance mechanisms for amyloid and other toxins along the perivascular pathways (Black et al., 2009; Gao et al., 2012). It is of note that pvWMH are also associated with CAA and visible as microbleeds, which would be consistent with disruption of the perivascular pathway, thereby exacerbating deposition of periarteriole Aβ (Pettersen et al., 2008).

CAA and AD associated arterial damage, as described above, would decrease the functioning of both perivascular and glymphatic drainage by impairing arterial pulsations (Carare et al., 2008; Dorr et al., 2012; Iliff et al., 2013b). Despite little Aβ deposition, the structural and functional integrity of venules and veins also deteriorate in AD, which may lead to further impairments in amyloid clearance (Revesz et al., 2003; Weller et al., 2009; Lai et al., 2015). The presence of atherosclerosis is common in patients with AD, causing further irregularities in vascularization including the thickening of the perivascular space, which likely decreases vascular associated Aβ clearance (Ross, 1993; Frink, 2002; Roher et al., 2003). One of the major hallmarks of perivascular clearance is that soluble Aβ deposits along capillaries and arteries as ISF drains (Hawkes et al., 2013, 2014). Since para-venous drainage out of the brain is proposed to occur in the glymphatic system, it is surprising that Aβ deposits are not abundantly seen along venules and veins (Revesz et al., 2003; Weller et al., 2009; Iliff et al., 2012). There are a couple of explanations for this observation. First, it is possible that the lack of smooth muscle cells on veins provide less substrates for Aβ to deposit on Weller et al. (2008). Secondly, peripheral monocytes have been reported to enter the lumen of veins, but not arteries, and clear soluble Aβ (Michaud et al., 2013a).

Healthy lifestyle interventions play an important role in the maintenance of the preceding Aβ clearance mechanisms. As discussed above, consistent exercise, diet and sleep contribute to proper structural and functional integrity of the vasculature, maintaining regular blood vessel pulsations, which are the driving force behind both the perivascular and glymphatic clearance mechanisms (Carare et al., 2008; Iliff et al., 2013b). Together these conclusions stress the importance of these lifestyle interventions in the maintenance of vasculature health, and subsequently in delaying the progression of AD.

## Conclusion and Future Perspectives

In this review article, we have summarized three different therapeutic approaches that rely on various combinations of physiological Aβ clearance mechanisms. We have highlighted some caveats to these approaches and attempted to highlight mechanisms that might function in synergy to remove toxic Aβ from the CNS as a disease-modifying therapy. The function of the Aβ clearance mechanisms, efflux at the BBB, catabolism within the CNS or drainage via either the perivascular drainage pathway or glymphatics, must be considered when designing new therapeutics and interpreting results from ongoing clinical trials, including the potential exacerbation of clearance mechanisms from small vessel arteriolar, capillary and venular disease. We propose that clinical trial failure may not be the sole result of an ineffective drug candidate or wrong patient population but may represent a lack of endogenous clearance mechanisms needed to support drug effects.

Although all cases of AD are characterized pathologically in the same manner, the cause of disease and co-morbidities that contribute to disease progression vary extensively between patients. In light of this, multi-modal therapeutic approaches may need to be considered, with an eye on personalized medicine to account for the variability in presentation of AD patients, including reliable quantification of subtypes of small vessel disease as well as patterns of gray and white matter atrophy, which is overlooked by many currently popular automatic pipelines. Combination therapies are already in practice for various diseases and thus may also be necessary for AD. Furthermore, the treatment strategies may need to vary depending on disease state or age of the individual at presentation, with the goal of treating the disease or delaying the onset.

## Conflict of Interest Statement

The authors declare that the research was conducted in the absence of any commercial or financial relationships that could be construed as a potential conflict of interest.

## Footnotes

^1^www.clinicaltrials.gov

^2^http://www.ttpharma.com

^3^http://www.alzforum.org

^4^www.clinicaltrials.gov
